# Brain network correlates of affective symptoms in aMCI

**DOI:** 10.1016/j.nbas.2024.100126

**Published:** 2024-10-14

**Authors:** Parnika P. Saxena, Adam Turnbull, Daniel Kim, Barbara Sommer, F. Vankee Lin

**Affiliations:** Department of Psychiatry and Behavioral Sciences, Stanford University, USA

## Abstract

Affective symptoms (i.e., depression, anxiety, and apathy) are the most prevalent subsyndrome of neuropsychiatric symptoms (NPS) in preclinical dementia, such as amnestic mild cognitive impairment (aMCI), and remain a challenge to understand and treat. The distressing nature of these symptoms and complexity of their concurrence and interaction necessitates improved understanding of their underlying neural correlates. We analyzed the relationships between functional brain topology (i.e., the way the brain’s functional network is organized to allow efficient communication between regions) and affective symptoms in aMCI using cross-sectional and longitudinal methods. The analyses demonstrated that increased clustering coefficient (CC) was related to lower baseline and greater decreases in affective symptoms, while higher participation coefficient (PC) was correlated with more severe baseline affective symptoms. These findings suggest that the brain losing the capacity to form segregated functional units may be related to prevalence and severity of affective symptoms seen in aMCI.

## Introduction

Amnestic mild cognitive impairment (aMCI) is the most common preclinical syndrome for AD, with memory deficits being its primary cognitive symptom [1], [2]. The presence of neuropsychiatric symptoms in cognitively normal older adults predicts rapid cognitive decline (see systematic review [3]). In aMCI, affective symptoms are the most prevalent neuropsychiatric symptoms, and can reach upwards of 90% and exacerbate the progression of aMCI to AD dementia [4], [5], [6]. Furthermore, AD pathologies, e.g., amyloid or phosphorylated tau, worsen affective symptoms [7], [8]. Finding ways to treat affective symptoms in aMCI is therefore not only important for improving quality of life in people with aMCI but may be an important avenue for slowing/preventing progression to dementia. To date, neuropsychiatric symptoms have only demonstrated response to selected pharmacological treatments with various issues related to safety [9], while emerging non-pharmacological interventions might be efficacious but need further confirmation [10]. Regardless, there is a need to identify novel therapeutic targets for affective symptoms in aMCI.

Determining the neural correlates of affective symptoms in aMCI may provide a means of understanding the mechanisms underlying these symptoms that could provide avenues for novel treatments or a means of predicting response to existing treatments. A review of 20 years of work linking neuropsychiatric symptoms to brain measures identified 283 significant relationships across 118 papers [11]. Affective symptoms, specifically apathy and depression, were the most commonly linked to the brain, along with delusions. Frontal regions such as the anterior cingulate cortex (ACC) and orbitofrontal cortex (OFC) were most commonly linked to these symptoms, however, the authors note significant heterogeneity in findings across studies and could only conclude that there are likely changes to fronto-temporal-subcortical networks. This is in line with findings seen throughout the brain-behavior literature where identifying consistent brain-behavior relationships at the regional level has been challenging [12]. Along with the fact that well-supported interventions that target specific brain regions is also lacking (with the one potential exception being transcranial magnetic stimulation to the dorsolateral prefrontal cortex in depression [13]), these difficulties have driven neuroimaging researchers to consider other approaches that attempt to understand and quantify how the brain functions as an interconnected network, rather than as a collection of independent functional units.

The brain exists as a network of nodes and edges, with anatomically distant regions being functionally connected in a way that allows for efficient information transfer with minimal wiring costs [14]. Efficient processing is facilitated by the existence of functionally segregated clusters of nodes that can work independently on specialized tasks, but that are interconnected by relatively few long-range edges that allow for integration of information across the whole network during more complex multimodal tasks. The proper functioning of this network, i.e., an appropriate balance of segregation and integration, has been shown to relate to a range of behaviors [2]: a loss of either segregation or integration is associated with both affective symptoms [3] and AD dementia [4]. Recent theories have proposed that baseline segregation may be a marker of neural plasticity [15], or the capacity to benefit from treatments targeting specific neural processes that may include those underlying neuropsychiatric symptoms. While we know of no studies to date looking at global brain segregation and integration in relation to neuropsychiatric symptoms in aMCI, there have been null findings in late life depression [16]. However, given that AD pathology is known to affect network hubs [17] that are critical to the integration-segregation balance, we expect that the mechanisms may not be the same for mood symptoms in aMCI. Brain segregation is known to decrease with normal aging [18] and has been associated with cognitive decline [19]: it has been proposed that brain segregation may also be a marker of cognitive reserve, allowing cognition to remain stable in the presence of AD pathology [20]. Although the previous literature has focused mostly on segregation, our previous studies have identified both segregation and integration as important markers of improvements in cognitive symptoms following non-pharmacological interventions [21], [22], in line with the idea that a good balance of segregation and integration is key to optimal neural processing and that improvements in these markers may reflect neural plasticity. One paper found that AD brain networks were characterized by lower integration and increased segregation [23], in line with the degradation of key hubs involved in linking distinct functional units, but a study in a larger population found the opposite effect: AD severity was related to reduced network segregation independently from normal aging [24]. Together, these findings suggest that brain network functioning is disrupted by AD pathology, but the implications for neuropsychiatric symptoms are currently unclear. In this paper, we examined whether differences in brain segregation and integration are associated with affective symptoms in aMCI to improve our understanding of the mechanisms underlying these symptoms as well as to guide future intervention research.

## Methods

### Design and participants

We used data from a recently completed pilot randomized control trial (preregistered as NCT04099524 at clinicaltrial.gov, with details in the primary report [25]). A sample of 40 older adults with aMCI (mean age=71.0, SD=7.0, 24 female) recruited from local memory clinics were randomly assigned to a 4-week intervention of cognitive training with or without (sham) concurrent transcranial direct current stimulation (tDCS); data were collected at baseline, post-intervention, and after one-month follow-up. Inclusion criteria: (1) consensus diagnosis of MCI due to AD (2011 NIA-AA diagnostic criteria: Montreal Cognitive Assessment; MoCA version 2 education-adjusted total score of 18 ≤ x ≤ 26; one standard deviation below age- and/or education-corrected population norms for Rey’s Auditory Verbal Learning Test Lists C&D; preserved activities of daily living via self-report version of the Activities of Daily Living-Prevention Instrument total score ≤ 30; and absence of dementia); (2) presence of two neuropsychiatric symptoms with informant-rated Neuropsychiatric Inventory Questionnaire (NPI-Q) severity sum score >=3, rated by comparing to 6 months ago to capture a worsening trajectory. Participants with MRI (e.g., pacemaker) or tDCS (e.g., history of seizures, repetitive motor conditions, skin condition or sensitivity) contraindications were excluded. The human subjects research procedure was approved by the university’s Research Subjects Review Board with written informed consent obtained from all participants. The current paper did not aim to examine whether the intervention (or intervention and active control) induced any changes in brain topology or affective symptoms, but to exploit variation in affective symptoms and brain topology over time to understand their relationship. Descriptives for the total sample and each group are shown in Table 1.

**Table 1:** Sample characteristics (N = 40).

	Total (n = 40)	Intervention (n = 20)	Control (n = 20)	Statistics*
Baseline variable				
Age, mean (SD)	71 (7.0)	70 (6.6)	73 (7.1)	t = -1.47, p = 0.15
White, n (%)	39 (97.5)	20 (100)	19 (95)	χ^2^ = 1.03, p = 0.31
Female, n (%)	24 (60)	14 (70)	10 (50)	χ^2^ = 1.67, p = 0.20
Years of education, mean (SD)	16 (2.9)	16 (3.3)	16 (2.6)	t = -0.99, p = 0.33
MoCA, mean (SD)	23.33 (2.57)	22.80 (2.33)	23.70 (2.68)	t = -1.05, p = 0.30
Neurodegeneration, mean (SD)	2.77 (0.14)	2.75 (0.12)	2.78 (0.16)	t = -0.74, p = 0.47
NPI-Full total-raw, mean (SD)	14.57 (13.74)	13.90 (11.64)	15.25 (15.84)	t = -0.31, p = 0.76
NPI-Full total-adjusted for caregiving distress, mean (SD)	2.48 (2.18)	2.64 (2.34)	2.34 (2.07)	t = -0.41, p = 0.68
Repeated variable				
Affective symptoms/ Participant reported mood composite, mean (SD)				
• baseline	0.13 (0.76)	0.20 (0.78)	0.06 (0.75)	t = 0.57, p = 0.58
• post-intervention	−0.01 (0.85)	−0.03 (0.91)	0.02 (0.81)	t** = -0.15, p = 0.88
• follow-up	−0.13 (0.70)	−0.10 (0.80)	−0.15 (0.60)	t** = 0.19, p = 0.85
Brain topology: whole-brain CC, mean (SD)				
• baseline	0.171 (0.027)	0.17 (0.03)	0.170 (0.021)	t = 0.38, p = 0.71
• post-intervention	0.170 (0.028)	0.17 (0.03)	0.167 (0.025)	t = 0.60, p = 0.28
• follow-up	0.170 (0.030)	0.17 (0.03)	0.165 (0.025)	t** = 0.98, p = 0.34
Brain topology: whole-brain PC, mean (SD)				
• baseline	0.231 (0.018)	0.232 (0.018)	0.231 (0.017)	t = 0.33, p = 0.75
• post-intervention	0.228 (0.017)	0.226 (0.015)	0.231 (0.019)	t = -1.02, p = 0.31
• follow-up	0.234 (0.017)	0.234 (0.017)	0.234 (0.018)	t** = 0.07, p = 0.95

MoCA = Montreal cognitive assessment; NPI = Neuropsychiatric Inventory-full; neurodegeneration was calculated as the Alzheimer’s disease cortical thickness score from structural MRI using a previously described procedure [26].

*df = 38 except specifically indicated; **df = 37.

### Measures

All measures are described in detail the intervention report [25]; we will briefly re-describe them in this section.

### Behavioral data: Affective symptoms

Affective symptoms were measured using three self-report mood-related questionnaires that probed recent affective symptoms: depression (Geriatric Depressive Scale; GDS-30); anxiety (State-Trait-Anxiety-Inventory; STAI-trait); and apathy (Apathy Evaluation Scale; AES). Total scores were z-transformed across timepoints and averaged to create a composite affective symptom score. Across all variables, higher scores indicated more severe affective symptoms. Given the improved sensitivity of self-report vs. informant-report measures in our previous studies [25], [27], we chose to focus on self-report questionnaires for the current analysis.

### Neuroimaging data: Brain topology indices

#### Acquisition

The MRI protocol was conducted using a Siemens 3T Prisma (VE11C) scanner equipped with a 64-channel head coil. Anatomical EPI MPRAGE scan (TR/TE=1400/2.34ms, slice thickness=1mm, resolution=1mm isotropic, 192 slices, PE acceleration=GRAPPA, FA=70°, fat suppression=OFF, orientation=sagittal, echo spacing=7ms, FOV=256mm) and resting-state (TR/TE=1010/44ms, slice thickness=2mm, resolution=2mm isotropic, R=1, SMS/MB acceleration factor=8, FA=70°, fat suppression=ON, orientation=transversal, echo spacing=0.56ms, FOV=256mm, acquisition matrix=128x128, 80 slices, 253 volumes) data were used in this study.

#### Preprocessing

Data were analyzed using adapted scripts [28] (https://github.com/adamgeorgeturnbull/BEEM), using functions from FSL v6.0.5.1 and AFNI v21.1.07. Preprocessing followed a standard pipeline: first four volumes dropped, motion correction (FSL MCFLIRT), distortion correction (FSL topup), slice-timing correction (FSL slicetimer), co-registration and normalization to MNI space (FSL FLIRT and FNIRT), simultaneous nuisance regression (9p nuisance regression model: six rigid body realignment parameters, global signal, average CSF, and average WM signal) and temporal filtering (bandpass 0.009–0.08 Hz), followed by spatial smoothing (FWHM 6mm), using AFNI 3dTproject. Timeseries extraction and FC matrix generation (including r-to-z transformation) were performed using Python 3 and the nilearn package (Version 0.9.1), using the AAL3 atlas [29]. Co-registration and normalization, as well as connectivity matrices, were visually inspected for artifacts or poor quality. One participant was excluded for motion exceeding 1mm mean RMS. Two participants were excluded for consistently poor co-registration and normalization.

#### Brain topology measures

Brain topology was quantified using the GRETNA toolbox with graph metrics of integration (i.e., participation coefficient) or segregation (i.e., clustering coefficient), using approaches in previous studies [21], [22]. Functional connectivity matrices were converted into undirected, weighted adjacency matrices, with negative weights set to 0. Networks were thresholded using consistency thresholding at a network density of 30%. *PC:* PC measures the proportion (*k_i,m_*: connections within network/k*_i_*_:_ total connections) of a node’s connections to nodes outside of its own network (**Eq.1**), with a range between 0 (no connections outside own network) and 1 (equal within- and between-network connections). We assigned nodes to the 7 Yeo networks [30] and calculated mean PC across all nodes. The Yeo networks are one of the most widely-used and well-established functional parcellations of the human brain. We chose to use these networks due to their robust nature and to improve comparability with other studies in the literature. *CC:* CC (C_i_) is calculated as the proportion of connections between the nodes within its neighborhood (all adjacent nodes) divided by the number of all possible connections between them (**Eq.2**). The term *g_i_* is the subgraph that contains the direct neighbors of node *i*, $t_{i}$ is the total number of connections in *g_i_*,*_._*$k_{g_{i}}$ is the number of nodes in *g_i_*, and $k_{g_{i}}\left(k_{g_{i}}-1\right)/2$ is the total number of all possible connections in *g_i_*. We averaged the mean CC of all nodes (N) to obtain global CC (C_global_) (**Eq.3**).

$PC_{i}=1-\sum_{m=1}^{M}{\left(\frac{k_{i,m}}{K_{i}}\right)}^{2}$ (1)

$C_{i}=\frac{t_{i}}{k_{\mathrm{gi}}\left(k_{\mathrm{gi}}-1\right)/2}$ (2)

$C_{\text{global}}=\frac{\sum_{i=1}^{N}C_{i}}{N}$ (3)

#### Analysis

We: (1) examined the relationship between baseline affective symptoms and brain topology: primary generalized linear models were based on the relationship between affective symptom composite score and whole-brain integration and segregation scores, taking brain topology variables as predictors and affective symptom composite score as outcome, controlling for the degree of head motion during the resting state scan. To assess the role of certain confounds in these analyses, we performed a separate analysis controlling for age, gender, and MoCA: the results did not differ significantly and are not reported; (2) examined the relationship between change of affective symptoms and brain topology: generalized estimating equation with AR(1) correlational matrix was conducted, taking time-dependent CC and PC as predictors, controlling for time-dependent head motion, time point (linear function through baseline, post-intervention, and follow-up), and group assignment (intervention vs. active control), and time-dependent affective symptom composite or individual symptoms as the outcomes.

## Results

### Baseline brain topology and mood symptoms

Lower PC and higher CC at baseline were significantly related to less severe baseline affective symptom composite. When examining mood symptoms separately, depression and apathy were significantly (or with a trend) related to both PC and CC, but not anxiety (Table 2a). Of note, when controlling for age, gender, and executive function, results remain similar.

**Table 2a:** Relationships between baseline brain topology and mood symptoms.

	PC		CC	
	B (SE)	Wald’s χ^2^ (p value)	B (SE)	Wald’s χ^2^ (p value)
Mood composite	17.74 (6.09)	8.49 (0.004)	−12.83 (3.94)	10.61 (0.001)
Depression	124.55 (39.25)	10.07 (0.002)	−80.14 (25.39)	9.96 (0.002)
Apathy	185.63 (70.09)	7.01 (0.008)	−86.95 (45.34)	3.68 (0.055)
Anxiety	145.94 (82.03)	3.165 (0.075)	−80.05 (53.07)	2.28 (0.13)

### Changes in brain topology and mood symptoms

Only increase in CC, but not change of PC, was significantly related to decreases in affective symptom composite. When examining mood symptoms separately, decreases in apathy were significantly related to both an increase in CC and a decrease in PC (Table 2b).

**Table 2b:** Relationships between changes of brain topology and mood symptoms.

Mood composite	2.66 (2.51)	1.12 (0.29)	−2.59 (1.32)	3.85 (0.050)
Depression	9.48 (18.32)	0.27 (0.61)	−16.45 (9.61)	2.93 (0.087)
Apathy	60.82 (24.96)	5.94 (0.015)	–32.62 (12.37)	6.96 (0.008)
Anxiety	−11.91 (33.73)	0.16 (0.72)	−5.10 (18.58)	0.08 (0.78)

## Discussion

We found that both baseline whole-brain integration and whole-brain segregation were significantly related to baseline mood symptoms in aMCI. Specifically, baseline integration calculated as PC was significantly related to more severe mood symptoms at baseline, while baseline segregation calculated as CC was significantly related to less severe mood symptoms at baseline. Only the CC finding was also significant when considering longitudinal data across three timepoints. When looking at symptoms in isolation, the baseline PC and CC results were most robust for depression and apathy: these results did not seem to be driven by anxiety. This was also true of longitudinal findings that were particularly strong for apathy and non-significant for depression and anxiety. These results add to the literature using measures of brain topology to understand Alzheimer’s disease. Given the spread of pathology via network hubs that are critical for maintaining the balance of integration and segregation, this approach seems very promising for understanding how pathology may cause a breakdown of overall brain topology that has profound effects on behavior. While previous studies have linked these changes, particularly reduced segregation, to cognitive decline[19], [24], we expand this literature to suggest that changes in brain topology may also be responsible for increases in mood symptoms early on in disease progression. This is important as mood symptoms are the most common type of neuropsychiatric symptoms in aMCI and are associated with significant distress to individuals at risk for dementia and their caregivers.

These findings contrast with null findings in late life depression [16], potentially suggesting that the mechanisms responsible for mood symptoms in those at risk for dementia differ from typically aging adults. This is intuitive given the large impact of AD pathology on network hubs that lead to significant changes in brain topology in AD [24]. Decreases in brain segregation that occur in normal aging [18] is also associated with cognitive decline [19]: stable segregation may therefore act to support cognition even in the presence of AD pathology [20]. Our results suggest that in individuals that maintain brain segregation, mood symptoms may also be less severe, suggesting maintained segregation could be a key goal for protecting both cognition and neuropsychiatric symptoms in early AD. This is in line with empirical findings that suggest maintained segregation may also confer increased plasticity that allows for greater improvements following interventions targeted at cognition [15], [21] that may also hold for interventions targeted at mood symptoms. Our finding that integration may be detrimental for mood symptoms may reflect the same processes that are reflected in these segregation findings. While normal brain function requires specific regions to be connected across networks to allow for integration, neurodegeneration can also cause increased integration when the structure of the brain breaks down and the modules become less clearly segregated. This would be reflected in both an increase in integration and decrease in segregation. Studies have found increased integration in AD in line with this idea [31], and this may explain why increased integration is related to more severe mood symptoms while the reverse is true for integration. However, it is important to note that other studies have found reduced integration in AD [23]: this may be driven by different metrics used. PC specifically measures the ratio of between to within network connections: a reduction in the overall structure of the brain would therefore result in increased PC (normal brain function is characterized by more within than between network connections). This is not necessarily true for other measures of brain integration that only look at between network connections.

There are important limitations to the current analysis. Firstly, the sample used in the current study is relatively small and homogenous in terms of ethnic/racial diversity. It will be important to confirm these findings in a larger sample and assess whether they generalize across racial/ethnic minority groups. Additionally, we do not have a healthy control group as a comparison. Differences in integration/segregation have been associated with behavioral changes in typically aging individuals [18], [19]; in this study it is unclear the extent to which these results reflect a continuum of associations across the aging spectrum or whether the relationships between integration and segregation are qualitatively different in aMCI compared to typically aging individuals. We also do not have the power to assess whether the absence vs. presence of these symptoms individually vs. together is associated with different brain network properties, but given the complex co-occurrence of these symptoms in aMCI this will be important for future studies in larger cohorts to assess.

Ethics approval statement

Human subjects research procedure was approved by the Research Subject Board at the University of Rochester.

Patient consent statement

Written informed consent was obtained from both participants and their informants.

Clinical trial registration

The clinical trial that these baseline data are part of is registered at clinicaltrial.gov record NCT04099524: https://clinicaltrials.gov/ct2/show/NCT04099524. Note that this study does not report on the intervention, only baseline and longitudinal data controlling for group assignment.

## CRediT authorship contribution statement

**Parnika P. Saxena:** Writing – review & editing, Writing – original draft, Conceptualization. **Adam Turnbull:** Writing – review & editing, Writing – original draft, Software, Formal analysis, Conceptualization. **Daniel Kim:** Writing – review & editing. **Barbara Sommer:** Writing – review & editing. **F. Vankee Lin:** Writing – review & editing, Supervision, Funding acquisition, Formal analysis, Conceptualization.

## Declaration of competing interest

The authors declare that they have no known competing financial interests or personal relationships that could have appeared to influence the work reported in this paper.

## Data Availability

Data are available via the NIMH Data Archive (NDA), miNDAR package 1205214, Host: mindarvpc.cqahbwk3l1mb.us-east-1.rds.amazonaws.com. See: https://nda.nih.gov/nda/access-data-info.html#cloud for instructions on how to access data. Raw fMRI data are not available due to an institutional policy aimed at protecting vulnerable populations (in this case individuals with aMCI and NPS). Researchers that want to access raw fMRI data will need to obtain institutional IRB and data user agreement approval.
